# Translating Human Prototype Liver Implant Technology from Academia to Industry for Third-Party Transplant and In Vivo Validation

**DOI:** 10.3390/cells15100905

**Published:** 2026-05-15

**Authors:** Dagmara Szkolnicka, Lydia González del Barrio, Carlos D. Quintana Calderón, Justyna M. Kowal, Shruthi Sampath, Giles Dudley, Joakim Sørensen, Allan E. Karlsen, David C. Hay

**Affiliations:** 1 BioInnovation Institute, Ole Maaløes Vej 3, 3, 2200 Copenhagen, Denmark; 2 Novo Nordisk Ltd., Novo Nordisk Park 1, 2760 Malov, Denmark; 3 Centre for Regenerative Medicine, Institute of Regeneration and Repair, College of Medicine and Veterinary Medicine, The University of Edinburgh, Edinburgh EH16 4UU, UK

**Keywords:** pluripotent stem cell, hepatocyte, endothelial cell, tissue engineering, liver disease, liver function, in vivo transplantation, regenerative medicine, technology translation, external validation

## Abstract

**Highlights:**

**What are the main findings?**

- Stem cell-derived somatic cells form vascularized liver tissue.
- Vascularized liver tissue displays mature function in vitro and in vivo.

**What are the implications of the main findings?**

- Stem cell-derived vascularized liver tissue can be scaled.
- Vascularized liver tissue is functional post transplantation in vivo.

**Abstract:**

Annually, there are more than two million deaths from liver disease. This is driven by organ inflammation and scarring, leading to a decline in function and regeneration. Frequently, this can develop into decompensated liver disease, resulting in the loss of physiological balance and toxin build-up within the body, with an increased risk of patient mortality. Currently, there are no approved medicines for the long-term treatment of liver cirrhosis. The only successful treatment option for end-stage liver disease patients is donor organ transplantation. However, patient requirement outstrips the number of donated organs. To address this bottleneck, researchers around the world have developed cell-based prototype systems to restore failing liver function, with some in clinical trials. Although significant progress has been made, no mainstream commercial liver assist products are available for routine clinical use. In this study we developed a stem cell-derived vascularized liver tissue implant prototype from pluripotent cells. The liver tissue was produced from a stem cell line that is banked at clinical grade, and displayed stable and mature liver function over a 6-week period in vitro. This included decreasing levels of the fetal marker, alpha-fetoprotein, when the serum albumin increased. This was further supported by stable alpha-1-antitrypsin secretion and cytochrome P450 function. Following the establishment of stable liver tissue, it was delivered as a cell product or attached to an electrospun polycaprolactone scaffold, to form a tissue implant. Next, cellular material was quality-controlled, and subsequently shipped to a contract research organization for external in vivo validation. The transplanted liver tissue functioned when implanted into the kidney capsule and subcutaneously, remaining functional for up to two weeks in vivo.

## 1. Introduction

Liver disease is a common source of patient morbidity and mortality. Key factors to progressive disease in humans are tissue inflammation and organ scarring [1]. To date, the clinical management of certain progressive and end-stage forms of liver disease is limited by the lack of scalable cell-based solutions [2]. The hepatocellular component of the liver performs hundreds of different functions, including the bio-transformation of toxic components so that they can be efficiently excreted from the body. When liver function drops below the critical threshold, waste products build up in the blood plasma. Although acellular liver dialysis systems have been used as a bridge to organ transplant, they have failed to show significant survival benefits in randomized clinical trials of advanced disease [3]. At present, donor liver transplantation is the only viable and successful treatment option for patients with critically failing liver function. However, organ donation is not scalable and therefore cannot meet clinical demand. Additionally, patients receiving donor organs are required to take lifelong immunosuppression to prevent organ rejection, which can have ‘off-target’ effects over time [4].

To try and address the bottleneck in delivering scalable liver support, researchers have focused on a number cell-based approaches, from differing tissue sources. This includes primary and stem cell-derived hepatocytes, liver organoids, chemically induced liver progenitors, macrophages and mesenchymal stromal cells [5]. Excitingly, some of those therapies are in clinical trials, showing early signs of safety and efficacy in patients with established cirrhosis [6]. We and others have focused on the use of pluripotent stem cells (PSCs) to build prototype human liver tissue for modeling human biology ‘in the dish’, and to support failing liver function in vivo [7]. The advantages of using such a system, with tissue self-assembly in microwell formats, includes the generation of renewable and programmable sources of human material for biomedical application, including drug testing, disease modeling and cell-based therapy. These have advantages over other methodologies that require the use of human donor material. However, to fully realize the potential of PSC-derived material, it is necessary to better define their manufacture, so that scalable and quality-controlled products can be tested and qualified by third parties, before moving toward large-scale manufacture and safety studies in humans.

With this in mind, the focus of our study was to translate a pioneering tissue engineering approach validated in a pre-clinical model of liver disease, developed in academia [8]. We secured access to a GMP grade hESC line, which was adapted to our defined extra-cellular matrix formulations, then expanded and cryo-banked at defined cell numbers for tissue engineering experiments. Post thaw, hESCs were expanded and driven toward hepatocyte and endothelial cell lineages using defined media formulations [9,10]. These procedures were developed in both hESC and iPSC lines, successfully generating stable liver tissue using both manual and semi-automated procedures [8,9]. Through the blending of laminin extra-cellular matrices, it was possible to deliver more rapidly maturing liver tissue that was stable and functional in nature. Following the process scale-up and tissue phenotyping, stem cell-derived vascularized liver tissue was shipped to a contract research organization for in vivo transplantation. In these studies, we detected the presence of circulating human serum albumin in mice, providing validation that stem cell-derived liver tissue can be produced at scale and shipped, and is functional after third-party transplantation in vivo.

In summary, we successfully developed a scalable human liver tissue product from human pluripotent stem cells. Vascularized liver tissue remained stable and functional in vitro for at least six weeks in vitro and two weeks post-transplant in vivo. Looking forward, we believe these studies serve as good platforms to build renewable and transplantable liver tissue, and have the potential to positively impact human liver disease treatments in the future.

## 2. Materials and Methods

### 2.1. Stem Cell Thaw and Expansion

Laminin-coated plasticware was prepared in line with experimental requirements 24 h prior to stem cell thaw. Laminins 521 and 111 were purchased from BioLamina (Sundbyberg, Sweden) and iMatrix-511 from Reprocell (Yokohama, Japan). The following day cryovials containing 1 × 10^6^ E1C3 hESCs were thawed and plated on the laminin-coated plasticware and maintained in mTeSR1 Plus™ (STEMCELL Technologies, Vancouver, BC, Canada). The cell seeding density in the different culture formats and the average stem cell number at 72 h post thaw on laminins are provided in Table 1 for reference. Cells were routinely passaged using Gentle Cell Dissociation Reagent (STEMCELL Technologies) and replated in antibiotic-free medium, as previously described [9].

### 2.2. hESC Hepatocyte Differentiation

For hepatic differentiation, E1C3 hESCs were dissociated using Gentle Cell Dissociation Reagent (STEMCELL Technologies) and plated onto pre-coated wells with recombinant laminins 521 and 111 (BioLamina) in mTeSR1 Plus™ supplemented with 10 μM Y-27632 (Bio-Techne, Minneapolis, MN, USA). To induce hepatocyte differentiation, E1C3 hESCs were plated at the densities shown in Table 2, and 24 h later hepatocyte differentiation was driven as previously described using the growth factors Wnt3a (Bio-Techne), Activin A (Bio-Techne), Hepatocyte Growth Factor (Fisher Scientific, Waltham, MA, USA), Oncostatin M (Fisher Scientific), and dimethyl sulfoxide (Sigma, Burlington, MA, USA) [9] [Supplementary Figure S2].

### 2.3. hESC Endothelial Cell Differentiation

Endothelial differentiation was performed using a published protocol [9,10]. Briefly, E1C3 hESCs were dissociated using Gentle Cell Dissociation Reagent (STEMCELL Technologies) and plated onto pre-coated wells with recombinant laminins 521 and 111 in mTeSR1 Plus™ supplemented with 10 μM Y-27632 (Bio-Techne). To induce endothelial differentiation, E1C3 hESCs were plated at the densities shown in Table 3, and 24 h later endothelial cell differentiation was driven using a small-molecule inhibitor of glycogen synthase kinase – 3 (Sigma-Aldrich, Burlington, MA, USA), bone morphogenetic protein 4 (Bio-Techne), StemPro-34 serum-free medium, vascular endothelial growth factor (Bio-Techne), and forskolin (Fisher Scientific), as previously described [9,10] [Supplementary Figure S3].

### 2.4. Vascularized Liver Sphere Self-Assembly in Microwell Culture and Phenotyping

To generate 3D liver spheres, day 9 stem cell-derived hepatic progenitors with day 5 endothelial cells were mixed at the ratio of 3.3:1, in an agarose microwell using the 3D Petri dish mold system (Sigma Aldrich, Burlington, MA, USA), as previously described [11]. Over a six-week period, the stem cell-derived liver sphere-conditioned medium was harvested and assayed for alpha-fetoprotein (AFP), albumin (ALB) and alpha-1 antitrypsin (A1AT) protein secretion using commercially available ELISA kits (AH Diagnostics, Tilst, Denmark and Abcam, Cambridge, UK), according to the manufacturer’s instructions. Three independent biological experiments were performed (*n* = 3). For each weekly collection, the culture medium was replaced with fresh growth factor-supplemented medium 24 h before sampling and incubated overnight at 37 °C. The conditioned medium was then collected and analyzed by ELISA. The secreted protein levels were normalized to the total protein content, and measured using a bicinchoninic acid (BCA) assay, as previously described [9]. Normalized ELISA data are presented as micrograms of secreted protein per milliliter of medium per milligram of total protein (μg/mL/mg). Cyp1A2 metabolic activity was measured using P450-Glo assay kit (Promega, Madison, WI, USA) according to the manufacturer’s instructions, as previously described [9]. Cyp1A2 activity was assessed in three independent biological experiments (*n* = 3). Briefly, Cyp1A2 substrate was added to fresh growth-supplemented culture medium at a final concentration of 100 μM and incubated with liver spheres for 24 h at 37 °C. Following incubation, 50 μL of conditioned medium from each well was transferred in technical triplicate to a 96-well plate and mixed with 50 μL of luciferin detection reagent. The samples were incubated for 20 min in the dark to stabilize the luminescent signal, which was then measured using luminometer and expressed as relative light units (RLU). Cyp1A2 activity was normalized to total protein content measured by BCA assay and presented as RLU per milliliter of medium per milligram of total protein (RLU/mL/mg). Variability between replicates is shown as standard deviation (SD) from three independent experiments. Data from week 1 and week 6 were compared using an unpaired *t*-test.

### 2.5. Preparation of Stem Cell-Derived Liver Tissue for Transplantation

#### 2.5.1. Preparation of Liver Spheres for Kidney Capsule Implantation

Two hundred and fifty six (256) pluripotent stem cell-derived liver spheres (day 7 of cell aggregation), each approximately 400 µm in size, were implanted under the kidney capsule of an immunocompromised NRG mouse. Prior to implantation, spheroids were allowed to settle, and the supernatant was gently removed from the microwells. Liver spheres were then washed with 100 µL of PBS (with Ca^2+^ and Mg^2+^). Subsequently, the PBS was aspirated and fresh PBS (with Ca^2+^ and Mg^2+^) was added to facilitate the transfer of the spheroids into the P60 tubing. Once inside the tubing, the cells were centrifuged at 400 RCF for 3 min to pellet them within the tubing for transplantation.

#### 2.5.2. Preparation of Liver Tissue for the Subcutaneous Transplantation

Two hundred and fifty six (256) pluripotent stem cell-derived liver spheres were transferred onto a 1 cm^2^ polycaprolactone (PCL) electrospun scaffold (Electrospinning Company Limited, Oxfordshire, UK). Liver sphere-loaded scaffolds were stabilized with ring retainers and maintained in a culture medium supplemented with growth factors. Scaffolds with or without liver cells were prepared in a 24-well plate using 1.5 mL of medium per well. The plates were kept in an incubator at 37 °C and 5% CO_2_ until use. On the day of implantation (day 0), PCL control- and cell-loaded scaffolds were gently washed twice with PBS containing Ca^2+^/Mg^2+^ at room temperature under sterile conditions before implantation.

### 2.6. Animal and Surgical Procedures

#### 2.6.1. Kidney Capsule Transplant

For the purpose of this study, NOD-Rag2-/-IL2rg-/- mice (NRG; *n* = 15) were used. The animals were anesthetized with isoflurane (2–4%) in an induction chamber before being moved to a nose cone with isoflurane (1.5–2%) for the surgery. Before incision, local analgesia (approximately 100 μL of a mixture of 0.8 mL bupivacaine (2.5 mg/mL) and 1 mL lidocaine (10 mg/mL)) was applied, the skin was washed with ethanol, and the shaved area was covered with a barrier drape. Then, a small incision was made through the skin and muscle on the left backside of the animal, and the kidney was exteriorized. The area and the kidney were kept moist using saline. Using a 30G needle, the kidney capsule was nicked, and a spatula was used to create a small fan-shaped pouch under the capsule covering approximately one third of the exposed side of the kidney. The plastic P60 tube containing the cells and the Hamilton syringe were then slowly screwed until the cells were transferred. After removing the tube, the hole in the kidney capsule was closed with tissue adhesive.

#### 2.6.2. Subcutaneous Transplant

The study was performed using NRG mice (*n* = 40). The animals were stratified into two groups: control (empty scaffold; *n* = 20) and experimental (liver sphere-loaded scaffolds; *n* = 20). The following procedure was performed under sterile conditions in a laminar airflow bench. Animals were anesthetized (isoflurane 2–4%, 100% O_2_) and placed in a supine position in a nose cone. The abdomen was shaved over a 3 × 3 cm area. The skin was sterilized with ethanol and covered with a barrier drape. A 1 cm incision was made in the skin, and a subcutaneous pocket was created on one side using blunt dissection. The plate containing the scaffold (approximately 1 cm^2^ in size) was removed from the incubator once the animal was anesthetized and the incision was ready for scaffold transplantation. Using forceps, the retainer was removed and the scaffold was carefully lifted to ensure it was held only at the edges. With the subcutaneous pocket held open, the scaffold was placed under the skin with the cells facing the abdominal wall. The incision was closed using wound clips and the animal was placed into a clean cage for recovery. The animals were kept on a heating mat throughout the entire procedure and were closely monitored until fully recovered from anesthesia.

### 2.7. In Vivo Testing

All in vivo studies were performed by Minerva Imaging ApS, Lyshøjvej 2, DK-3650 Ølstykke. They are licensed by the Danish Animal Experimentation Inspectorate and conducted the studies in accordance with the Danish Animal Experimentation Act (Bekendtgørelse af lov om dyreforsøg BEK nr 1065 af 25/09/2024). The Danish act is stricter than and fully compliant with the European directive (2010/63/EU) and with internationally accepted principles for the care and use of laboratory animals. Furthermore, all work in the animal facilities follows standards and classifications set by relevant authorities including the Danish Working Environment Authority, the Danish Environmental Protection Agency, and the Danish Health Authority for Radiation Protection.

In the case of kidney capsule studies, blood sampling was performed by perforation of the sublingual vein, and blood was collected directly into lithium heparin tubes. Plasma samples for human albumin analysis were collected on day 7 and 14 (for other time points see Supplementary Figure S4). To evaluate human albumin concentrations, most plasma samples were diluted 1:4 and spiked with 2.25 ng/mL recombinant human albumin to bring concentrations within the ELISA kit range (Abcam; ab179887). Standard curves were generated as described in the manufacturer’s protocol, with the addition of a 1:4 dilution of naïve plasma from age- and strain-matched mice.

Plasma samples from mice transplanted with the PCL scaffold were collected via the sublingual vein on day 1, 3, 7, 14, and 28 post scaffold implantation, and via cardiac puncture at day 56, for the analysis of human albumin (see Supplementary Figure S4 for days 14, 28 and 56). To evaluate human albumin concentrations, plasma samples were diluted at 1:32 (day 1 and 3 samples) or 1:4 (day 7–56 samples) ratios and spiked with 3 ng/mL recombinant human albumin to bring concentrations within the kit range (Abcam; ab179887). Samples were normalized to control (empty scaffold) samples from the corresponding day. The variability between replicates is shown as standard error (SEM).

### 2.8. Body Weight Measurement

Animal body weight was monitored twice per week. Data are shown as mean ± SEM for all groups. All appropriate safety and welfare measures were taken into account. When there was uncertainty as to whether an animal had reached the predefined humane endpoint, the mice were evaluated by a veterinarian. The humane endpoint was defined as body weight loss exceeding 20%, at which point euthanasia was required. All animal experiments were conducted under a license approved by the National Animal Experiments Inspectorate under the Ministry of Environment and Food of Denmark. 

## 3. Results

### 3.1. Stem Cell Expansion and Differentiation

We sourced a hESC line that has been banked at clinical grade. The E1C3 line was seeded at the appropriate cell density (Table 1) then expanded in vitro on three recombinant extra-cellular matrix formulations: iMatrix-511, Laminin 521 and Laminin 521:111. During cell expansion, E1C3 hESCs remained pluripotent, expressing Oct4, Sox2 and Nanog, which was determined by immunostaining (Supplementary Figure S1). For scale-up experiments, hESCs were expanded on laminin 521 frozen down at 10^6^ per vial. Upon resuscitation for differentiation experiments, hESCs were plated in flasks pre-coated with recombinant laminin 521 or laminin 521 and 111. For hepatocyte differentiation, E1C3 hESCs were replated at the appropriate cell number (Table 2). For endothelial cell differentiation, E1C3 hESCs were replated at the appropriate cell number (Table 3). To induce hepatic differentiation, we used a stagewise use of factors and additives including Wnt3a, Activin A, Hepatocyte Growth Factor, Oncostatin M, and dimethyl sulfoxide (Supplementary Figure S2). To induce endothelial cell differentiation, we used the stagewise use of a small-molecule inhibitor of glycogen synthase kinase-3, bone morphogenetic protein 4, StemPro-34 serum-free medium, vascular endothelial growth factor, and forskolin (Supplementary Figure S3). As previously described, cell commitment to the hepatic lineage was assessed by cells positive for HNF4a and AFP (Supplementary Figure S2) [9], whereas endothelial cell commitment was confirmed by VE-Cadherin immunopositivity and MACS enrichment (Supplementary Figure S3) [9].

### 3.2. Vascularized Human Liver Tissue Development

**Figure 1:**
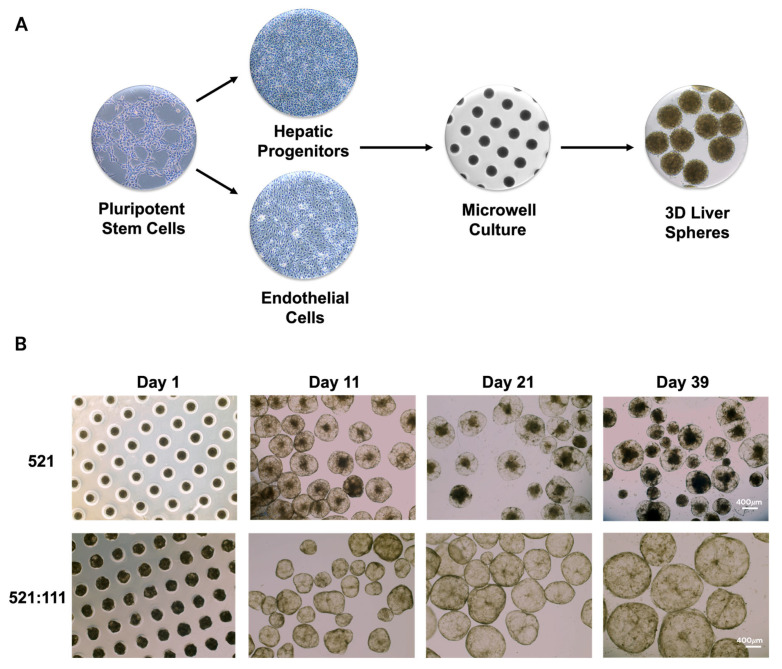
Liver tissue engineering. (**A**) Schematic representation of 3D liver spheres generated from hPSCs. (**B**) Brightfield microscopy images showing liver sphere morphology. Day 1 shows spheres growing inside the microwells. Subsequent days show growth of free-floating spheres in non-adhesive wells coated with poly 2-hydroxylethyl methacrylate (poly-HEMA). Days indicate the duration of sphere maintenance after cell aggregation. Spheres were generated from 2D cells cultured either on laminin-521 (LN521) alone or on a mixture of laminins (LN521:111). Scale bar: 400 µm.

Stem cell-derived progenitors and endothelial cells differentiated on laminin 521 and laminin 521:111-coated surfaces displayed similar morphologies and immunoreactivity. Following characterization, somatic cell populations were enzymatically removed from monolayer culture, combined at the ratio 3.3 (hepatic progenitors): 1 (endothelial cells), seeded into microwell culture at 1 million cells per 256-well micromold (Figure 1A) and left to self-assemble into three-dimensional aggregates. Liver sphere size increased over time, from 200 to 500 μM. During this period, liver spheres displayed mature function and phenotypic stability (Figure 1B). Stem cell-derived liver spheres derived on the laminin 521:111 matrix blend displayed superior characteristics to their 521 counterparts (Figure 1B). The liver spheres generated on laminin 521 became progressively granular in appearance (Figure 1B), with potentially necrotic aggregate formation over time. Therefore, we opted not to take the 521 procedures forward to the in vivo studies, due to their potential to cease function prematurely upon transplant.

### 3.3. Human Liver Tissue Phenotyping

**Figure 2:**
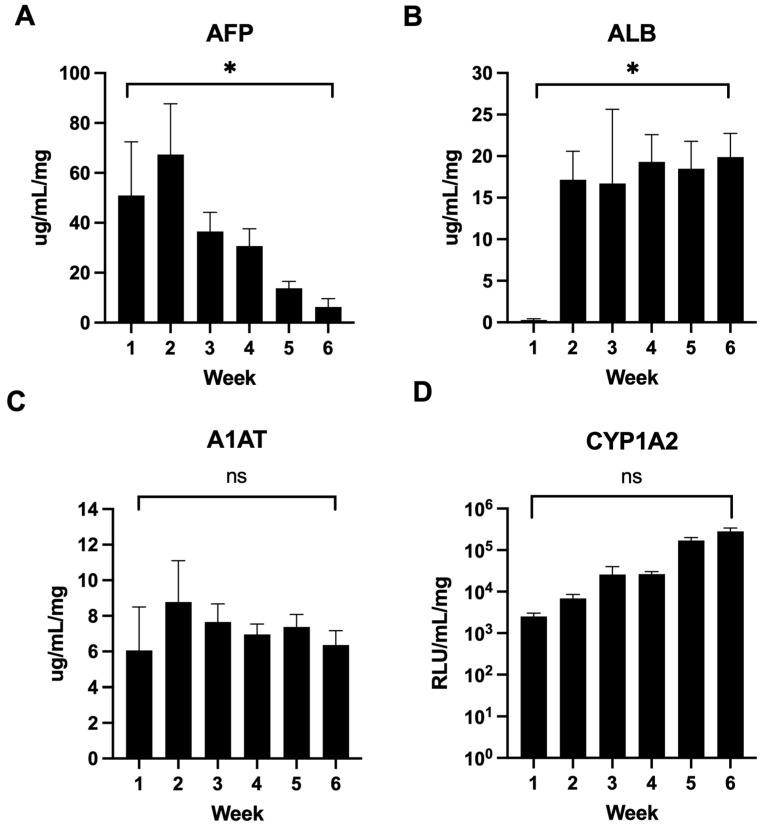
Liver tissue phenotyping. Secretion of the serum proteins. (**A**) Alpha-fetoprotein (AFP), (**B**) albumin (ALB), and (**C**) alpha-1 antitrypsin (A1AT) were measured by ELISA at the indicated time points post-agreggation (mean ± SD, *n* = 3). (**D**) Cytochrome P450 1A2 (Cyp1A2) activity was analyzed at the indicated time points during liver sphere culture (mean ± SD, *n* = 3). Data were compared between week 1 and 6 and analyzed by unpaired *t*-test: * (*p* < 0.05); ns (non-significant).

Next, laminin 521:111-generated liver spheres were phenotyped over a 6-week period. We tested AFP secretion as a marker of immaturity, albumin and Cyp1A2 function as markers of maturity, and alpha-1-antitrypsin which is expressed in both the fetus and adult. We observed a significant decrease in AFP secretion over the 6-week time course (from >50 μg/mL to <7 μg/mL) (Figure 2A). As AFP decreased, we observed a consequent increase in albumin secretion (from baseline to ~20 μg/mL) (Figure 2B). Throughout the differentiation time course, alpha-1-antitrypsin secretion remained stable (between ~6–8 μg/mL) (Figure 2C) and there was a non-significant trend to increased Cyp1A2 enzyme activity (from 10^3^ to 10^5^ RLU/mg/mL), demonstrating liver sphere maturation and phenotypic stability (Figure 2D).

### 3.4. Human Liver Tissue Transplantation In Vivo

**Figure 3:**
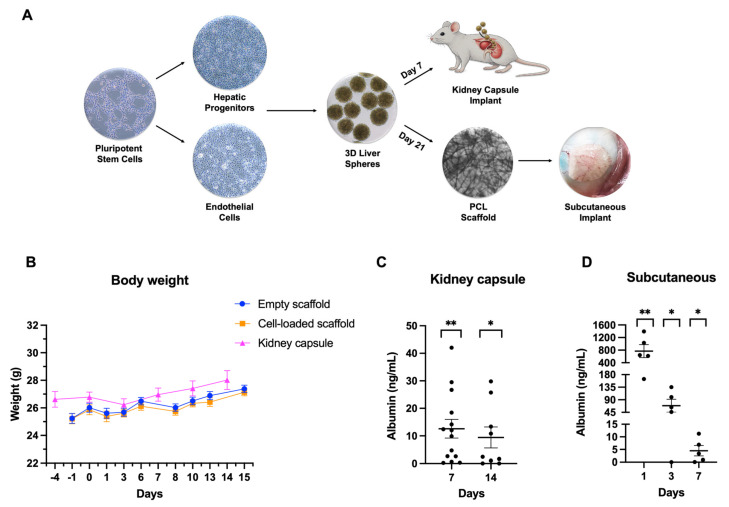
In vivo transplantation. (**A**) Schematic of 3D sphere preparation for two administration routes (kidney capsule transplantation and subcutaneous implantation). (**B**) Body weight (g) from transplantation to termination; day 0 denotes the day of transplantation. Data are shown as mean ± SEM for all three groups. (**C**) Plasma human albumin concentrations measured on days 7 and 14 post-implantation. Values at each point were compared with controls using an unpaired *t*-test: * (*p* < 0.05); and ** (*p* < 0.01). The graph shows individual values with mean ± SEM. (**D**) Plasma albumin levels measured by ELISA in animals one, three and seven days after cell-loaded scaffold implantation. Samples were spiked with recombinant albumin and corrected to control-group samples on the corresponding day; control-group values were subtracted from experimental-group values. Experimental and control groups were compared using Student’s unpaired *t*-test: * (*p* < 0.05); ** (*p* < 0.01). Bars represent mean ± SEM.

Following successful liver differentiation experiments in vitro, we scaled up the process for the in vivo studies. Following tissue engineering, stem cell-derived liver tissue was shipped to a contract research organization for transplantation and monitoring. The focus of the experiments was to assess stem cell-derived liver tissue ability to produce human proteins in vivo. To test this, we opted for two routes of administration in vivo. In total, 256 stem cell-derived liver spheres harvested at day 7 were produced (Figure 3A) and transplanted into the kidney capsule of immunocompromised (NRG) mice using established procedures developed at the contract research organization. Day 7 was chosen as the liver spheres were in the size range of 400–500 μM, and they could be delivered using a catheter into the kidney capsule. Cell transplantation proceeded well and liver spheres were well-tolerated, with no significant differences in animals’ body weight or behavior versus the controls (Figure 3B). Human albumin secretion was significantly increased over the controls, circulating at >9 ng/mL for at least 2 weeks post-transplant (Figure 3C; for the remaining samples see Supplementary Figure S4). Next, we compared the kidney capsule route of administration to subcutaneous implantation. For these experiments we delivered day 21 liver tissue on PCL electrospun scaffolds (Figure 3A). The scaffolds were well-tolerated with no significant differences found in animals’ body weight or behavior versus the control group (Figure 3B). Additionally, the implants were well vascularized and did not display any sign of tissue rejection or scarring even after 2 months of implantation (Figure 3A, subcutaneous implant). Liver tissue implants contributed to circulating albumin levels. This was significantly increased over the control group, with ~770 ng/mL detected on day 1, ~70 ng/mL on day 3, and ~5ng/mL by day 7. Albumin levels reached baseline by day 14 (Figure 3D; for the remaining samples see Supplementary Figure S4).

## 4. Discussion

Chronic and end-stage liver diseases affect approximately 1.5 billion people worldwide. Current therapeutic approaches are limited by organ availability and potential complications [4]. Therefore, cell-based artificial liver support and therapies have been developed as alternatives to organ transplant. They have shown potential to promote liver regeneration and repair in pre-clinical models and, excitingly, some are moving into advanced-stage clinical trials [5,6].

In addition to the use of primary cells, pluripotent stem cells have emerged as a promising renewable source of human hepatocytes for therapy [5,7]. Pluripotent stem cells are capable of generating all the somatic cell types found in the human body and can be scaled and differentiated to demand, removing the need for organ donation. At present, there are approximately 115 pluripotent stem cell-based clinical trials operating internationally, targeting a range of diseases, including liver disease, thereby demonstrating their strong potential for future human disease management [12].

We and others have developed highly efficient procedures to produce human hepatocytes at scale for modeling human health and diseases ‘in the dish’ [13,14,15,16], as well as in relation to transplanting them in vivo to provide support for damaged and failing liver function [7,8]. The ability to produce large quantities of cells under chemically defined conditions, from pluripotent stem cells, is a distinct advantage of this technology, and key to the production of GMP-grade material for safety and efficacy in human clinical trials. To stabilize derivative liver tissue phenotype, it has been necessary to employ tissue engineering processes, incorporating the different cell types found in the human liver [9,17]. This not only provides trophic support for hepatocytes but also captures elements of the liver structure that are essential for normal liver function in vivo [13].

The purpose of our study was to take a prototype technology from academia and create a defined manufacturing process to produce human vascularized liver tissue at the necessary scale for in vivo testing. In particular, it was important to blend two different recombinant laminins (521 and 111) when generating vascularized liver spheres to prevent cell necrosis. The reason for necrotic core formation in liver spheres, composed of cell types generated on laminin 521 only, is unknown. However, it is interesting to note that stem cell-derived hepatocytes produced on laminin 521 and 111 displayed a more mature phenotype than their laminin 521 counterparts [18]. Following liver tissue engineering, stem cell-derived liver tissue was shipped to a contract research organization for in vivo transplantation and the external validation of human function. Stem cell-derived liver tissue was transplanted into both the kidney capsule and subcutaneously, and demonstrated stable human albumin production for at least 2 weeks in vivo. These data, in combination with other cell-based therapy studies within the field, highlight the promise that PSCs have to offer liver disease patients. That being said, there are significant limitations that must be overcome. These include cell engraftment efficiencies, the longevity of the graft function, and the interaction with the host’s immune system. Therefore, future cell-based therapies, in combination with new biomaterials, advanced gene-editing tools, and in vivo pre-conditioning, will be essential to deliver off-the-shelf human liver tissue that is suitable for new therapeutic strategies [19,20].

The goal of these studies was to develop implantable liver tissue that avoids the hostile environment of the abdominal cavity in late-stage liver disease patients [21], and can be delivered to patients using local anesthetic to reduce the need for complex surgery [22]. Therefore, the subcutaneous compartment, to deliver ectopic liver support, is currently our favored implant site. However, the current decline in liver function observed in vivo is an important consideration. Going forward, we will focus on enhancing liver tissue stability following transplant, with the aim of delivering more durable graft function and remote liver support. This will require further optimization of both the in vitro and in vivo niches, with programmable biomaterials likely to play a key role [23].

## 5. Conclusions

In conclusion, we have successfully translated a prototype liver therapy from academia to industry. To move this toward future clinical testing, we have significantly modified cell specification and tissue engineering processes to generate human vascularized liver tissue that can be delivered in vivo by a third party. The transplanted human liver tissue was functional, with circulating human albumin detected for at least 2 weeks post-transplant. Importantly, these studies complement other areas of focus in the field which include cell scale-up, tissue conditioning, in vivo transplantation, tissue recellularization, organ bioprinting, immune cell therapy, and the standardization of clinical trials [24,25,26,27,28]. Looking forward, it is important to test the safety and efficacy of stem cell-derived liver tissue implants in further animal models; however, we believe that this study represents a strong platform to build on, in the quest to treat critically failing liver function in humans.

## 6. Patents

Stimuliver ApS has one patent application: Endothelial cell differentiation EP4549549A1.

## Figures and Tables

**Table 1:** Seeding densities for E1C3 hESCs using mTeSR1 Plus™ and laminin coatings.

	6 WP (per well)	T12.5	T25	T75	T175
Cell seeding	230.000	300.000	600.000	1.8 × 10^6^	4.2 × 10^6^
Cells number (×10^6^) 72 h	1.3–2.1	1.5–2.5	3–5	10–15	23–35
mTeSR1 Plus (mL)	2	2.5	5	15	35

**Table 2:** Seeding densities for E1C3 hESCs for hepatocyte differentiation.

	6 WP (per well)	T12.5	T25	T75	T175
Cells number (×10^6^)	0.384	0.5	1	3	7
Volume (mL)	2	2.5	5	15	35

**Table 3:** Seeding densities for E1C3 hESC endothelial cell differentiation.

	6 WP (per well)	T12.5	T25	T75	T175
Cell number (×10^6^)	0.288	0.375	0.75	2.25	5.25
Volume (mL)	2	2.5	5	15	35

## Data Availability

The full data obtained in vitro and in vivo are presented graphically within the article.

## Supplementary Materials

The following supporting information can be downloaded at: https://www.mdpi.com/article/10.3390/cells15100905/s1. Figure S1: hESC culture and pluripotency marker expression on different human laminin matrices; Figure S2: hESC differentiation to hepatic progenitors on different human laminin matrices; and Figure S3: hESC differentiation to endothelial cells on different human laminin matrices. Figure S4: Body weight and human albumin secretion in the remaining in vivo samples.

## Abbreviations

The following abbreviations are used in this manuscript:

PSC	Pluripotent Stem Cell
hESC	Human Embryonic Stem Cell
GMP	Good Manufacturing Process
AFP	Alpha-Fetoprotein
HNF4a	Hepatic Nuclear Factor 4 Alpha
ELISA	Enzyme-Linked Immunosorbent Assay
PCL	Polycaprolactone
3D	3-Dimensional
CRO	Contract Research Organization
