# Supplementary material for: Translating Human Prototype Liver Implant Technology from Academia to Industry for Third-Party Transplant and In Vivo Validation
Source: Cells. 2026 May 15;15(10):905. doi: 10.3390/cells15100905 (PMC13204467; doi:10.3390/cells15100905)
Supplement: Supplementary file 1 [file cells-15-00905-s001.zip › cells-4280170-supplementary.pdf]

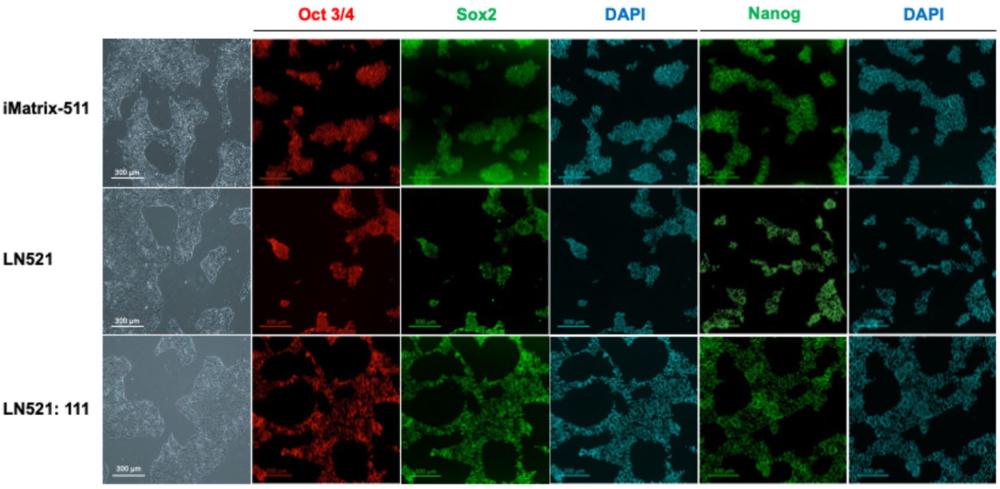

Supplementary Figure S1: hESC culture and pluripotency marker expression on different human laminin matrices. Phase-contrast images of representative fields of view of hPSCs, and immunostaining for pluripotency markers (OCT3/4, SOX2, NANOG) in hPSCs cultured on iMatrix-511, laminin-521 (LN521), or a mixture of laminins (LN521:111). DAPI represents a negative control. Scale bar: 300 µm.

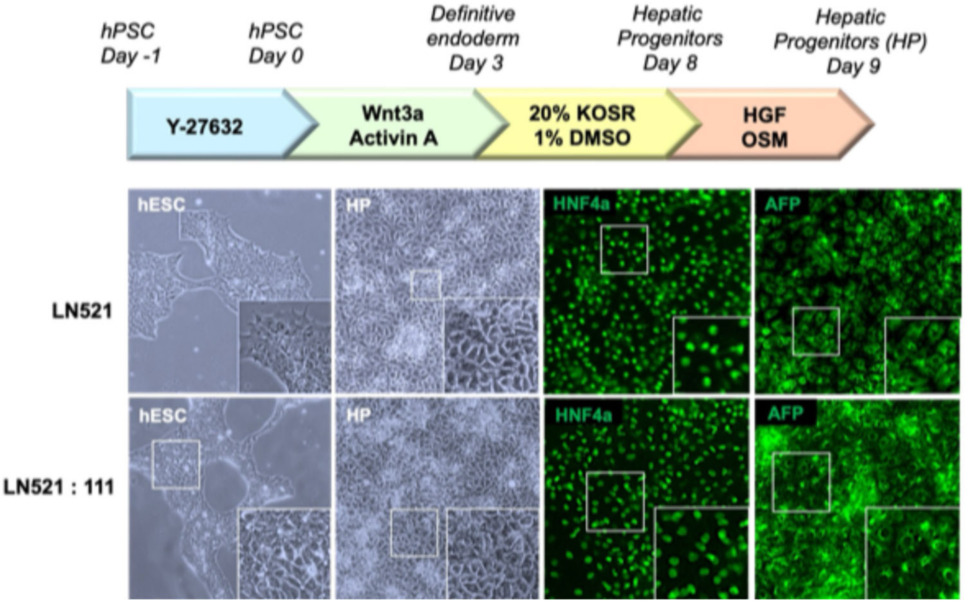

Supplementary Figure S2: hESC differentiation to hepatic progenitors on different human laminin matrices. Schematic representation of the protocol used to differentiate hESCs into hepatic progenitor cells (HP). Phase-contrast images of representative fields of view at the hPSC stage and at the hepatic progenitor stage (HP), together with immunostaining of day 9 HPs showing expression of HNF4α and AFP. Magnification x10. White squares indicate zoom of a specific area. KOSR – knockout serum replacement, DMSO - dimethyl sulfoxide, HGF – hepatocyte growth factor, and OSM – oncostatin M.

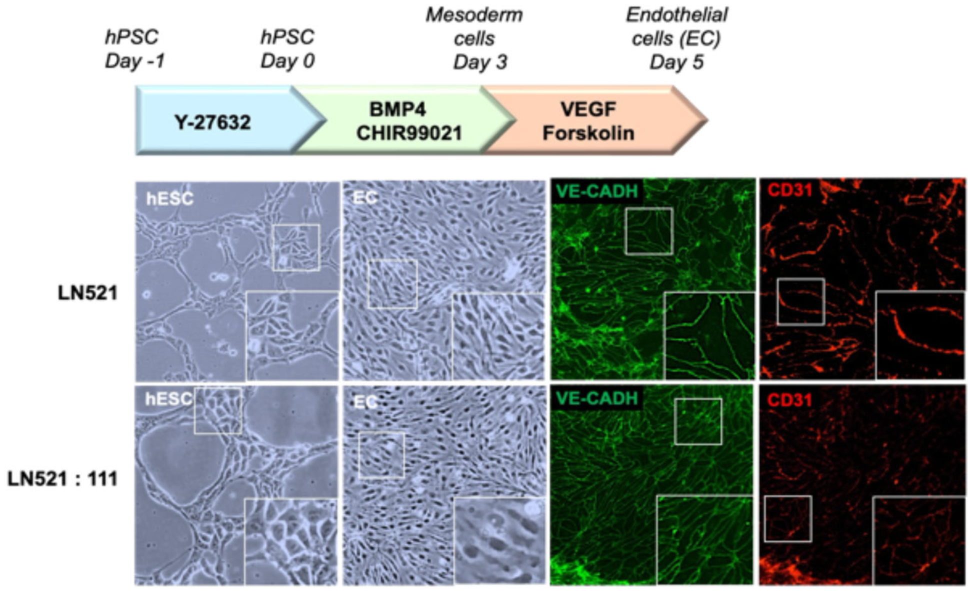

Supplementary Figure S3: hESC differentiation to endothelial cells on different human laminin matrices. Schematic representation of the protocol used to differentiate hPSCs to endothelial cells (EC). Phase-contrast images of representative fields of view at the hPSC stage and at the endothelial cells stage (EC), together with immunostaining of day 5 ECs showing expression of VE-cadherin (VE-CADH) and CD31. Magnification x10. White squares indicate zoom of a specific area. BMP4 - Bone morphogenic protein 4, and VEGF - vascular endothelial growth factor

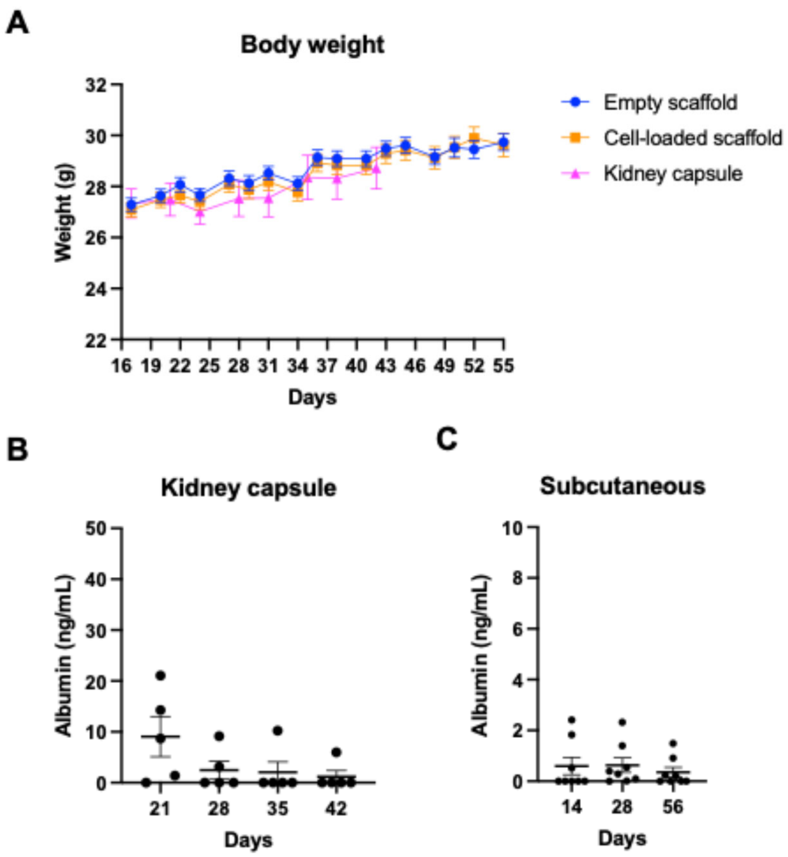

Supplementary Figure S4: Human albumin secretion in vivo. (A) Body weight (g) measured from day 17 until termination (kidney capsule – day 42; subcutaneous – days 55). Data are shown as mean ± SEM. (B) Plasma human albumin concentrations measured on days 21, 28, 35 and 42 post-implantations. A spiked concentration of 2.25ng/mL human albumin was subtracted from all values. The graph shows individual values with mean ± SEM. Data were analyzed by un-paired t-test; no significant differences were detected. (C) Plasma albumin measured by ELISA in animals 14, 28, and 56 days after scaffold implantation. Control-group samples were subtracted from experimental-group samples. Separate comparisons were performed for each day. Data were analyzed by unpaired t-test (control vs experimental); no significant differences were detected. Graph show mean ± SEM.
